# Neural correlates of reduced depressive symptoms following cognitive training for chronic traumatic brain injury†

**DOI:** 10.1002/hbm.24052

**Published:** 2018-03-23

**Authors:** Kihwan Han, David Martinez, Sandra B. Chapman, Daniel C. Krawczyk

**Affiliations:** ^1^ Center for BrainHealth, School of Behavioral and Brain Sciences The University of Texas at Dallas Dallas Texas; ^2^ Department of Psychiatry University of Texas Southwestern Medical Center Dallas Texas

**Keywords:** depression, intervention, mental health, neural marker, neuroplasticity

## Abstract

Depression is the most frequent comorbid psychiatric condition among individuals with traumatic brain injury (TBI). Yet, little is known about changes in the brain associated with reduced depressive symptoms following rehabilitation for TBI. We identified whether cognitive training alleviates comorbid depressive symptoms in chronic TBI (>6 months post‐injury) as a secondary effect. Further, we elucidated neural correlates of alleviated depressive symptoms following cognitive training. A total of seventy‐nine individuals with chronic TBI (53 depressed and 26 non‐depressed individuals, measured using the Beck Depressive Inventory [BDI]), underwent either strategy‐ or information‐based cognitive training in a small group for 8 weeks. We measured psychological functioning scores, cortical thickness, and resting‐state functional connectivity (rsFC) for these individuals before training, immediately post‐training, and 3 months post‐training. After confirming that changes in BDI scores were independent of training group affiliation, we identified that the depressive‐symptoms group showed reductions in BDI scores over time relative to the non‐depressed TBI controls (*p *<* *.01). Within the depressive‐symptoms group, reduced BDI scores was associated with improvements in scores for post‐traumatic stress disorder, TBI symptom awareness, and functional status (*p *<* *.00625), increases in cortical thickness in four regions within the right prefrontal cortex (*p*_vertex _< .01, *p*_cluster_<.05), and decreases in rsFC with each of these four prefrontal regions (*p*_vertex _< .01, *p*_cluster _< .0125). Overall, these findings suggest that cognitive training can reduce depressive symptoms in TBI even when the training does not directly target psychiatric symptoms. Importantly, cortical thickness and brain connectivity may offer promising neuroimaging markers of training‐induced improvement in mental health status in TBI.

## 1 INTRODUCTION

Depression is the most frequent psychiatric condition reported among individuals with traumatic brain injury (TBI), up to 53% within the first year of an injury (Bombardier et al., 2010). Depression is a complex disorder. Depressive symptoms develop based on abnormal interactions among genes, neurotransmitter systems, brain circuitry, and social factors (Kupferberg, Bicks, & Hasler, 2016). Accordingly, biological factors such as pre‐injury susceptibility and injury locations at the acute stage of TBI are related to the development of depression after TBI (Jorge et al., 1993). Psychosocial factors also contribute to the occurrence of depression in individuals with TBI. TBI‐induced changes in physical attributes, levels of cognitive functioning, and personality often result in a gradual loss of friendships and increased chances of social isolation (Dawson & Chipman, 1995; Hoofien, Gilboa, Vaki, & Donovick, 2001; Paniak, Phillips, Toller‐Lobe, Durand, & Nagy, 1999). These limited social opportunities further reduce successful social interactions, increasing the likelihood of experiencing depressive symptoms (Ylvisaker, Turkstra, & Coelho, 2005). Overall, negative cycles of reduced social interactions and brain dysfunction lead to greater chances of experiencing depression among individuals with TBI. Further, comorbid depression in TBI can impact other domains such as cognitive functioning (Rapoport, McCullagh, Shammi, & Feinstein, 2005), quality of life (Bombardier et al., 2010), functional disability level (Fann, Katon, Uomoto, & Esselman, 1995), suicidality (Silver, Kramer, Greenwald, & Weissman, 2001), and recovery (Mooney, Speed, & Sheppard, 2005), which reciprocally increases the occurrence of depressive symptoms in TBI.

Psychological intervention (Clark & Beck, 2010) is one of the promising options to treat depression in individuals with TBI, though more research is needed to provide stronger evidence for its efficacy (Fann, Hart, & Schomer, 2009; Gertler, Tate, & Cameron, 2015). Cognitive behavioral therapy (CBT) is a popular psychological intervention for depression. CBT provides a set of cognitive strategies to directly manage negative emotion (Beck, Rush, Shaw, & Emery, 1979; Beck & Freeman, 1990). Several studies in TBI reported reduced depressive symptoms following CBT (Ashman, Cantor, Tsaousides, Spielman, & Gordon, 2014; D'Antonio, Tsaousides, Spielman, & Gordon, 2013; Fann et al., 2015; Ponsford et al., 2016; Tiersky et al., 2005; Topolovec‐Vranic et al., 2010). However, psychological intervention approaches are multifaceted in nature, exhibiting improvements in other domains, for example, primarily cognition, then depression secondarily. Since depression influences cognition and other outcomes following TBI (Bombardier et al., 2010; Rapoport et al., 2005), it would be informative to identify which specific areas of functional improvement are directly related to reduced depression severity after intervention. Further, little is known about underlying brain‐based changes associated with reduced depression severity following psychological interventions for TBI. As depression alters brain structure and function (Mayberg, 1997; Price & Drevets, 2012), elucidating the neural mechanisms by which interventions reduce depressive symptoms could allow us to better understand the effects of cognitive interventions for depression in TBI.

Brain morphometry and resting‐state functional connectivity (rsFC) are of particular interest to elucidate the underlying neural plasticity associated with reduced depressive symptoms following interventions. Brain morphometry quantifies gray matter volume, cortical thickness, and the shape of subcortical regions (Ashburner & Friston, 2000; Dale, Fischl, & Sereno, 1999; Fischl, Sereno, & Dale, 1999; Patenaude, Smith, Kennedy, & Jenkinson, 2011). Morphometry measures have revealed altered brain structure following depression (Peterson et al., 2009; Schmaal et al., 2017), TBI (Turken et al., 2009), and comorbid depressive symptoms in TBI (Hudak et al., 2011). Resting‐state functional MRI (rsfMRI) reveals the organization of intrinsic functional networks (Biswal, Yetkin, Haughton, & Hyde, 1995). Previous rsFC studies have demonstrated abnormal intrinsic functional networks after depression (Wang, Hermens, Hickie, & Lagopoulos, 2012), TBI (Sharp, Scott, & Leech, 2014), and comorbid depressive symptoms in TBI (Han, Chapman, & Krawczyk, 2015). In the context of experience‐dependent neuroplasticity in adults, both morphometry and rsFC are also valuable measures (Guerra‐Carrillo, Mackey, & Bunge, 2014; Kelly & Castellanos, 2014; May & Gaser, 2006). Relevant prior findings include increases in cortical thickness after memory training for the elderly (Engvig et al., 2010) and changes in gray matter volume following balance training for Parkinson's disease (Sehm et al., 2014). Previous studies also reported resting‐state network changes following motor training for healthy individuals (Lewis, Baldassarre, Committeri, Romani, & Corbetta, 2009), cognitive rehabilitation for multiple sclerosis (de Giglio et al., 2016), and cognitive therapy for depression (Beevers, Clasen, Enock, & Schnyer, 2015; Li et al., 2016).

Previously, we reported the efficacy of a strategy‐based reasoning intervention for chronic TBI, compared to an information‐based intervention (Vas et al., 2016), and the underlying neuroplasticity following intervention (Han, Davis, Chapman, & Krawczyk, 2017). Both intervention programs in our previous study included social interactions in small group settings with regular meetings comprised of multiple individuals with TBI and social contact with instructors who fostered an empathetic environment for the participants along with cognitive activities. In light of previous studies about the impacts of social interaction on depression (Ashman et al., 2014; Cruwys et al., 2013; Cruwys et al., 2014) and neural effects of cognitive intervention on depression (Beevers et al., 2015; DeRubeis, Siegle, & Hollon, 2008; Li et al., 2016), our intervention study design provided an opportunity to characterize the impact of reduced depressive symptom severity on other psychological functioning scores and the neural correlates of reduced depressive symptoms following cognitive intervention for TBI.

In the current study, we assessed whether small‐group‐based cognitive interventions reduce depressive symptom severity in individuals with TBI as a secondary effect. Further, we sought to identify the effects of reduced depressive symptoms severity on other psychological functions. Lastly, we utilized cortical thickness and rsFC to examine the neural plasticity associated with reduced depressive symptom severity following cognitive interventions. We hypothesized that small‐group‐based TBI cognitive interventions would reduce depressive symptoms severity in individuals who reported depressive symptoms at baseline relative to individuals with minimal‐to‐no depressive symptoms. We further hypothesized that such reductions in depressive symptoms following cognitive training would induce changes in cortical thickness and rsFC in individuals after TBI.

## 2 MATERIALS AND METHODS

We conducted a two arm, double‐blind (i.e., types of intervention were blinded to participants and data acquisition team) randomized control study with a 3‐month follow‐up phase (see (Krawczyk et al., 2013) for detailed methods). The presence of depressive symptoms was blinded to intervention instructors. We acquired participants’ depressive symptom severity, psychological functioning scores, and MRI data prior to training (TP_1_), after training (TP_2_), and 3 months later (TP_3_).

### 2.1 Participants

We analyzed 79 individuals (age 20–65) at the chronic stage of TBI (>6 months post‐injury) ranging from lower moderate disability to lower good recovery on the Extended Glasgow Outcome Scale (GOS‐E) (Wilson, Pettigrew, & Teasdale, 1998). Fifty‐seven out of the 79 participants completed MRI scans and passed the quality assurance (QA) procedures described below. In our previous studies, we demonstrated that a subset of the current sample sustained TBI‐related abnormalities in neuropsychological performance, rsFC, and white matter integrity, relative to healthy individuals (Han et al., 2015; Han, Chapman, & Krawczyk, 2016). We recruited our sample from the Dallas–Ft. Worth community and conducted a phone‐screen interview before inclusion. The inclusion criteria for the participants specified (1) a GOS‐E score between four and seven, consistent with the likely ability to successfully engage in training programs, (2) the presence of TBI identified by the TBI screening form (Corrigan & Bogner, 2007), (3) >6 months post‐injury time at the beginning of training, (4) age between 19 and 65 years old for the duration of the study, (5) the ability to understand, read, and speak English, (6) no history of significant clinically‐diagnosed neurological or psychiatric co‐morbidities other than self‐reported depressive symptoms, (7) no current use of any prescription medications that may have affected depressive symptoms or cognitive performance, and (8) not currently pregnant. The primary TBI mechanisms of the participants included blasts, blunt force trauma, falls, athletic impacts, vehicular accidents, or combinations thereof. *Initial* injury severity was *retrospectively estimated* utilizing the Ohio State University TBI identification method (OSU TBI‐ID; Corrigan & Bogner, 2007). The rationale for utilizing the OSU TBI‐ID is described in detail in our previous study (Han et al., 2017). Both civilians and military veterans were included (See Table 1 for demographics). We confirmed that no participants showed visible focal lesions, contusions, mass shifting, or extreme cortical thinning on structural MRI, therefore minimizing the potential effects of macro structural injuries on preprocessing for cortical surface reconstruction and rsFC analyses. All participants provided written informed consent, and this study was conducted in compliance with the Declaration of Helsinki. The study was approved by the Institutional Review Boards of the University of Texas at Dallas and University of Texas Southwestern Medical Center.

**Table 1:** Participant demographics by group after quality assurance procedures

Demographics	TBI‐plus‐depressive symptoms^a^	TBI‐only^b^	*p* values	Effect sizes^f^
The number of participants (SMART, BHW)	30, 23	10, 16	>0.1	0.48
Age (years)^c^	41.9 ± 14.0	40.4 ± 12.8	>0.1	0.05
Education (years)^c^	16.1 ± 2.9	15.6 ± 2.1	>0.1	0.05
Current IQ^c^ ^,d^	109.4 ± 9.3	110.6 ± 12.8	>0.1	−0.11
Premorbid IQ^c^, ^d^	110.8 ± 8.1	110.8 ± 8.7	>0.1	<0.01
Gender (male, female)	33, 20	15, 11	>0.1	0.61
Civilians, Veterans	34, 19	19, 7	>0.1	1.52
Post‐injury time (years)^c^	9.1 ± 9.8	9.1 ± 6.3	>0.1	−0.14
Estimated injury severity (mild, moderate, severe)^e^	36, 5, 12	17, 4, 5	>0.1	0.09
Primary cause of injury (blast, blunt force trauma, fall, athletic impacts, vehicle accidents, combined)	4, 9, 8, 11, 17, 4	5, 4, 3, 4, 6, 4	>0.1	0.23

a BDI–II of 14–63 at baseline.

b BDI–II of 0–13 at baseline.

c Mean and standard deviation values were reported.

d Current IQ and premorbid IQ were estimated from the Wechsler Abbreviated Scale of Intelligence and Wechsler Test of Adult Reading, respectively.

e Based on the OSU TBI screening form (Corrigan & Bogner, 2007).

f Rank‐biserial correlation for age, education, premorbid IQ, and post‐injury time; Hedge's g for current IQ; Cramer's V for estimated injury severity and primary cause of injury; Odds ratio for the other demographics.

Abbreviations: SMART = Strategic Memory Advanced Reasoning Training; BHW = Brain Health Workshop; IQ = intelligent quotient.

### 2.2 Experimental design

All participants were randomly assigned into one of the two training groups: (1) a strategy‐based reasoning training called Strategic Memory Advanced Reasoning Training (SMART; *n *=* *40) or (2) the information‐based training called Brain Health Workshop (BHW; *n *=* *39). Both training programs comprised of 12 sessions (1.5 hr per session) for 8 weeks with quizzes, homework assignments, and projects conducted in small group settings (4–5 participants) that involved social interactions. The SMART group focused on selective attention, abstract reasoning and other thinking strategies (Vas, Chapman, Cook, Elliott, & Keebler, 2011), and the BHW group focused on education regarding brain anatomy, function, the effects of TBI, neural plasticity, and the effects of diet, sleep, stress, social activity, and exercise on the brain performance (Binder, Turner, O'connor, & Levine, 2008). More specifically, the SMART group was trained to (1) manage information by blocking distractions and irrelevant information by avoiding multitasking, (2) increase the ability to understand general ideas and extract take‐home messages from information, and (3) interpret information from divergent perspectives. This set of strategies was aimed at enhancing cognitive control over information. The participants practiced learned strategies using news articles and audio‐video clips. The BHW group was not trained on any strategies to improve cognition, but rather was provided with information to engage with to serve as an active control. Both programs were led by two trained clinicians. Participants were also encouraged to discuss the application of learned information to their daily lives. To match the effects of social activities between the two groups, we maintained comparable numbers of participants for each training group during training sessions. Both training programs were conducted at The University of Texas at Dallas Center for BrainHealth^®^. See (Vas et al., 2016) for more detailed descriptions of the SMART and BHW programs.

### 2.3 Assessment of depressive symptoms

Depressive symptom severity was quantified using the Beck Depression Inventory‐II (BDI–II; Beck, Steer, & Brown, 1996). According to BDI scoring guidelines for identifying the presence of depressive symptoms (Beck et al., 1996), we subdivided TBI participants into two groups using baseline total BDI scores: those with TBI plus mild‐to‐severe depressive symptoms (*n *=* *53; BDI = 14–63) and those with TBI only/minimal depressive symptoms (*n *=* *26; BDI = 0–13). The cutoff score for the presence of depressive symptoms was based on the suggested total BDI score for a diagnosis of depression from the BDI‐II manual (Beck et al., 1996).

Since the two BDI‐based groups participated in one of the two training programs, the participants were divided into four groups: (1) TBI‐plus‐depressive symptoms who participated in the SMART, (2) TBI‐plus‐depressive symptoms who participated in the BHW, (3) TBI‐only who participated in the SMART, and (4) TBI‐only who participated in the BHW (see Table 2). We first set up a full model including interaction terms between training types and the presence of depressive symptoms, and performed linear mixed effects model (LME; Bernal‐Rusiel, Greve, Reuter, Fischl, & Sabuncu, 2013) analysis of total BDI scores using a piece‐wise linear model with a break‐point at TP_2_ and a randomly varying intercept. We utilized the LME model because it is more appropriate for longitudinal data with increased sensitivity, specificity and reliability than other alternatives such as repeated‐measures ANOVA (Bernal‐Rusiel et al., 2013).

**Table 2:** The sample size (SMART, BHW) and timing of psychological functioning assessments and MRI scans per time point by group

Data type	Time point	TBI‐plus‐depressive symptoms	TBI‐only	Weeks from baseline
Psychological functioning scores	TP_1_	53 (30, 23)	26 (10, 16)	–
	TP_2_	43 (26, 17)	24 (9, 15)	8.8 ± 0.8
	TP_3_	42 (22, 20)	22 (8, 14)	18.2 ± 1.7
Structural MRI scans^a^	TP_1_	36 (22, 14)	21 (7, 14)	–
	TP_2_	30 (20, 10)	17 (5, 12)	8.9 ± 0.8
	TP_3_	23 (13, 10)	16 (5, 11)	20.6 ± 1.4
Resting‐state fMRI scans^a^	TP_1_	30 (19, 11)	18 (7, 11)	–
	TP_2_	26 (17, 9)	15 (4, 11)	8.9 ± 0.8
	TP_3_	20 (11, 9)	14 (4, 10)	20.6 ± 1.5

a Only MRI scans that passed the quality assurance procedures were reported.

Abbreviations: TP_1_ = within one month prior to training; TP_2_ = immediately after training completed; TP_3_ = 3 months after training completed.

Using the full model, we confirmed that there were no statistically significant interactions between training types *and* the presence of depressive symptoms for temporal changes in total BDI scores. This was presumably due to the reduced sample size for each of the cells (see Table 2). To obtain a parsimonious statistical model, we thus performed the LME analysis of a simplified model with group membership based on either training types only or the presence of depressive symptoms only. This procedure was aimed to determine whether training‐induced changes in total BDI scores were specific to exclusively training types *or* the presence of depressive symptoms.

### 2.4 Psychological functioning

We selected a subset of psychological functioning scores that were affected by depressive symptom severity in individuals with TBI in our previous study (Han et al., 2015) and additional survey‐based functional outcome measures that are known to be related to depressive symptoms (Goverover & Chiaravalloti, 2014; Hudak, Hynan, Harper, & Diaz‐Arrastia, 2012; Malec, Testa, Rush, Brown, & Moessner, 2007). The utilized psychological functioning scores included inhibition/switching of the color‐word test and category switching of the verbal fluency test, both from the Delis‐Kaplan Executive Function System (Delis, Kaplan, & Kramer, 2001), immediate recall and delayed recall from the Wechsler Memory Scale‐Fourth Edition (Wechsler, 2008), the Posttraumatic stress disorder Check List Stressor‐specific (PCL‐S) (Weathers, Litz, Herman, Huska, & Keane, 1993), and the Satisfaction With Life Scale (Diener, Emmons, Larsen, & Griffin, 1985). The additional survey‐based functional outcome measures included TBI Awareness Questionnaire (Sherer, 2004), and Functional Status Examination (FSE; Dikmen, Machamer, Miller, Doctor, & Temkin, 2001). We administered the survey‐based measures using the corresponding forms on the same day that we assessed the participants’ psychological functioning. We also acquired full scale intelligent quotient‐2 (FSIQ‐2) from the Wechsler Abbreviated Scale of Intelligence for estimated current IQ (Wechsler, 1999), and FSIQ from the Wechsler Test of Adult Reading for estimated premorbid IQ (Wechsler, 2001).

### 2.5 MRI data acquisition

Participants underwent structural MRI scans in a Philips Achieva 3T scanner. In each imaging session, T1‐weighted sagittal magnetization prepared rapid acquisition gradient echo (MPRAGE) images were acquired using a standard 32‐channel head coil (repetition time (TR)/echo time (TE) = 8.1/3.7 ms; flip angle (FA) = 12°; field of view (FOV) = 25.6 × 25.6 cm; matrix = 256 × 256; 160 slices, 1.0 mm thick). In this imaging session, one or two 416‐s runs of rsfMRI scans were also acquired with the same head coil using a $T_{2}^{*}$‐weighted image sequence (TR/TE = 2,000/30 ms; FA = 80°; FOV = 22.0 × 22.0 cm; matrix = 64 × 64; 37 slices, 4.0 mm thick). During rsfMRI acquisition, the participants were asked to remain still with their eyes closed. Early stage observations revealed that the QA procedures with only one rsfMRI run yielded high rates of participant exclusion. Thus, we acquired two rsfMRI runs for the remainder of data collection. Refer to the rsfMRI data analysis section for our strategy to account for differences in total number of rsfMRI scans across participants.

### 2.6 Cortical thickness analysis

#### 2.6.1 Cortical surface reconstruction and cortical thickness measurement

We reconstructed the cortical surface from each MRI scan with FreeSurfer v.5.3.0 (http://surfer.nmr.mgh.harvard.edu/). The cortical surface reconstruction procedures from T1‐weighted MPRAGE images included nonuniform intensity correction, volumetric transformation to Talairach space, intensity normalization, skull stripping, segmentation of white and gray matter structures, tessellation of the gray/white matter boundary, smoothing of the gray/white matter boundary, surface inflation, topology correction, spherical mapping, and spherical registration. The gray/white matter boundary and pial surfaces were placed at the locations where the greatest intensity gradient occurred. More technical details of cortical surface reconstruction procedures have been described elsewhere (Dale et al., 1999; Fischl et al., 1999; Fischl, 2012). We obtained cortical thickness as the closest distance between the estimated gray/white matter boundary and pial surfaces at each vertex across the cerebral cortex (Fischl & Dale, 2000).

#### 2.6.2 Longitudinal analysis of cortical thickness associated with BDI

To obtain a reliable longitudinal analysis of cortical thickness, we utilized the longitudinal processing stream (Reuter, Schmansky, Rosas, & Fischl, 2012) in FreeSurfer. This included skull stripping, Talairach transforms, atlas registration, and spherical surface maps and parcellations initialized from an unbiased within‐subject template (Reuter, Rosas, & Fischl, 2010). For the group analysis, we resampled cortical thickness for each of the scans on a standard template, followed by surface smoothing with a 10 mm full‐width‐at‐half‐maximum (FWHM) Gaussian kernel.

**Figure 1:**
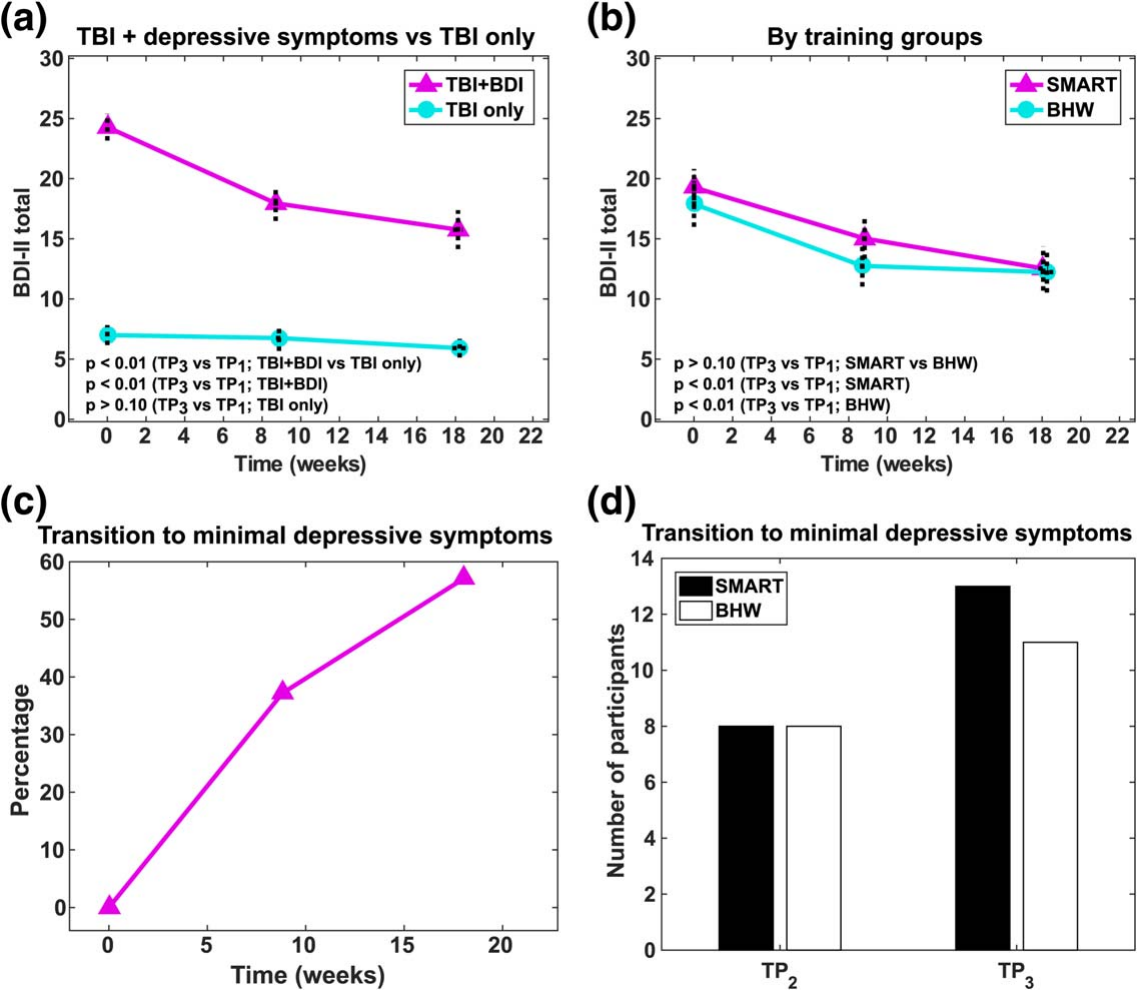
Temporal changes in depressive symptoms severity. (a) Changes according to the presence of depressive symptoms. (b) Changes according to the types of intervention program. (c) Percentage of participants with resolved depressive symptoms following intervention. (d) The number of participants with resolved depressive symptoms following training, according to the types of intervention program. BDI–II, Beck Depression Inventory‐Second Edition; TBI + BDI, TBI‐plus‐depressive symptoms group (BDI–II of 14–63 prior to intervention); TBI only, TBI with minimal depressive symptoms group (BDI–II of 0–13 prior to intervention) [Color figure can be viewed at http://wileyonlinelibrary.com]

We performed the LME analysis on the preprocessed longitudinal data using a piece‐wise linear model with a break‐point at TP_2_, randomly varying intercept, age covariate, and between‐ and within‐subject BDI covariates for each BDI group. Specifically, the cortical thickness of subject *i* at time point *j*, *y_ij_*, can be written as:

$$\begin{array}{c} y_{\text{ij}}{=\beta }_{1}{+\beta }_{2}\cdot t_{\text{ij}}{+\beta }_{3}\cdot \left(t_{\text{ij}}-\tilde{t}\right)\cdot H\left(t_{\text{ij}}-\tilde{t}\right){+\beta }_{4}\cdot S_{i}{+\beta }_{5}\cdot S_{i}\cdot t_{\text{ij}}{+\beta }_{6}\cdot S_{i}\cdot \left(t_{\text{ij}}-\tilde{t}\right)\cdot H\left(t_{\text{ij}}-\tilde{t}\right)\\ {+\beta }_{7}\cdot A_{i}{+\beta }_{8}\cdot D_{i}\cdot {\bar{B}}_{i}{+\beta }_{9}\cdot D_{i}\cdot \left(B_{\text{ij}}-{\bar{B}}_{i}\right){+\beta }_{10}\cdot N_{i}\cdot {\bar{B}}_{i}{+\beta }_{11}\cdot N_{i}\cdot \left(B_{\text{ij}}-{\bar{B}}_{i}\right){+b}_{i}{+e}_{\text{ij}} \end{array},$$

where *t_ij_* is the time of measurement for subject *i* at time point *j*, $\tilde{t}$ is an average time of measurement at TP_2_, *S_i_* is an indicator function for the SMART group for subject *i*, *A_i_* is the age for subject *i*, ${\bar{B}}_{i}$ is the average BDI score over time for the subject *i*, *B*_ij_ is the BDI score for subject *i* at time point *j*, *D_i_* is an indicator function for the TBI‐plus‐depressive symptoms group for subject *i*, *N_i_* is an indicator function for the TBI‐only group for subject *i, b_i_* is a subject‐specific intercept (cortical thickness of subject *i* at TP_1_), *e_ij_* is the measurement error for subject *i* at time point *j*, and *H*(·) represents the Heaviside step function. In this LME model, $\beta_{1},\ldots ,\beta_{6}$ account for training‐specific temporal changes in cortical thickness, which are covariates of no interest in this study. We included BDI covariates according to the BDI‐based group membership as temporal changes in total BDI scores were only depend on the presence of depressive symptoms at TP_1_ (see result section and Figure 1).

We performed subsequent statistical inferences for the within‐subject BDI covariates for the BDI groups (i.e., $\beta_{9}$ and $\beta_{11}$). Each of the statistic maps were then thresholded at *p*_vertex _< .01 and *p*_cluster _< .05 to identify cortical regions that showed statistically significant associations between temporal changes in cortical thickness and temporal changes in total BDI scores in each of the BDI groups.

### 2.7 rsfMRI data analysis

#### 2.7.1 Volumetric rsfMRI preprocessing

Volumetric rsfMRI data were preprocessed in a subject‐native space in AFNI (Version AFNI_17.3.02; Cox, 1996). For each rsfMRI run, the initial four frames were discarded, followed by despiking, slice timing correction, motion correction, spatial resampling (4 mm isotropic), normalization to whole‐brain mode of 1,000, band‐pass filtering (0.009 < *f* < 0.08 Hz), and linear regression. In the linear regression, we detrended the rsfMRI time series (third order) and included nuisance variables for the six rigid body motion profiles. Additional nuisance variables included cerebrospinal fluid (CSF) and white matter signals that were obtained from averaging over the lateral ventricles and deep cerebral white matter, respectively. We also included the first temporal derivatives of the aforementioned parameters (i.e., the motion profiles, and the average CSF and white matter signals). After the linear regression, we performed motion “scrubbing” (Power, Barnes, Snyder, Schlaggar, & Petersen, 2012) with a framewise displacement (FD) of 0.5 mm and a standardized DVARS to prevent potential motion artifacts (van Dijk, Sabuncu, & Buckner, 2012; Power et al., 2012; Satterthwaite et al., 2012). If two runs of rsfMRI scans were acquired, we temporally concatenated them. To account for the differences in total number of frames after motion scrubbing across rsfMRI scans, all remaining frames were trimmed to a minimum length (121 frames; 242 s) across all rsfMRI scans as suggested in (Power et al., 2014). Note that we performed spatial registration to a template and spatial smoothing after volume‐to‐surface mapping for the fMRI data. Refer to the next section for details.

#### 2.7.2 Surface mapping

Using FreeSurfer, we projected the preprocessed volumetric functional time series and subject masks onto mesh surfaces of each subject by averaging the time series across the voxels along the line between two matching nodes of the white and pial surfaces, followed by resampling onto the same standard template that was used for the cortical thickness analysis and surface smoothing at 10 mm FWHM. We also obtained an intersection mask for the mesh nodes in which rsfMRI signals existed across all subjects and time points.

#### 2.7.3 Longitudinal analysis of seed‐based connectivity associated with BDI

Our previous study demonstrated that brain regions where training‐induced cortical thickness changes occurred also impacted corresponding resting‐state functional connectivity (Han et al., 2017). Thus, we selected four seed regions where statistically significant associations between temporal changes in cortical thickness and total BDI scores occurred, and we assessed their seed‐based connectivity. These four seed regions were the right ventrolateral prefrontal cortex, anterior prefrontal cortex, and dorsal prefrontal cortex within Brodmann Area 9 and Brodmann Area 8B (see the results section). We obtained the seed regions from the standard template that was used for the cortical thickness analysis. With these seeds we obtained seed‐based connectivity for each of the scans, followed by a Fisher's *Z*‐transform and scaling to *Z*‐scores.

We performed the LME analysis with the same model used for the cortical thickness analysis, excluding the age covariate (after confirming no significant effects of age on rsFC). In this LME analysis, we assessed associations of training‐related changes in rsFC to changes in total BDI–II scores. We also assessed relationships between rsFC and each of the Buckley three‐factor scores (Buckley, Parker, & Heggie, 2001) because of reported sensitivity of rsFC to the subtypes of depressive symptoms (Han et al., 2015). The Buckley three‐factor model decomposes the total BDI‐II scores into cognitive, affective, and somatic symptoms (Buckley et al., 2001). The cognitive factor includes items regarding sadness, pessimism, past failure, guilty feelings, punishment feelings, self‐dislike, self‐criticalness, suicidal ideation, and worthlessness. The affective factor includes items probing loss of pleasure, crying, loss of interest, and indecisiveness. The somatic factor includes the agitation, loss of energy, sleep disturbance, irritability, appetite disturbance, concentration difficulty, fatigue, and loss of sexual interest test items. The Buckley factor model provides a better characterization of depressive symptom severity of psychiatric patients over alternative models (Vanheule, Desmet, Groenvynck, Rosseel, & Fontaine, 2008) and is the best model for veterans with polytrauma (Palmer et al., 2014). Statistically significant associations were identified at *p*_vertex _< .01 and *p*_cluster _< .0125(=.05/4) to correct for multiple comparisons across vertices and the number of seeds.

### 2.8 Quality assurance

Only participants who passed the following quality assurance procedures were included in analysis: less than 5 missed training sessions, no significant brain atrophy on structural MRI scans, a minimum of 4 min of rsfMRI volumes after motion scrubbing (van Dijk et al., 2010), and acquisition timing of MRI scans or psychological functioning scores within the two‐standard‐deviation band from the mean at each time point. Further, we excluded MRI scans from the LME analysis if corresponding BDI scores were not available. See Table 2 for the number of MRI scans that survived QA procedures.

### 2.9 Statistical analyses

All statistical analyses were conducted in MATLAB R2013a. First, we performed the Shapiro‐Wilk normality test on demographic data (age, years of education, current IQ, premorbid IQ, and post‐injury time) within each group at α = 0.05. Age, years of education, premorbid IQ, and post‐injury time did not pass the Shapiro‐Wilk normality test. Thus, the Mann‐Whitney *U* test was used to compare these demographics between the groups. We performed *t* tests for group comparisons of current IQ. The Fisher's exact test was used to compare the groups according to gender distributions, proportion of civilians and veterans, and proportion of SMART and BHW participants. We used the likelihood ratio chi‐square test to compare the distribution of estimated initial injury severity and primary cause of injury between the groups. Similar to the analyses of cortical thickness and rsFC, we performed the LME analysis on psychological functioning scores. In these analyses, we additionally included a years‐of‐education covariate for age‐adjusted scores from the color‐word and verbal fluency tests. Both age and education covariates were included for immediate and delay recall scores. For scores from the PCL‐S, TBI awareness questionnaire, FSE, and satisfaction with life scale, we did not include age and education covariates, and found no statistically significant effects for these measures.

We also obtained effect sizes for demographics, and correlations between reduced depressive symptoms and psychological functioning, cortical thickness and resting‐state functional connectivity, respectively. For the group demographic comparisons we obtained Hedge's *g* for the *t* test, rank‐biserial correlation for the Mann‐Whitney *U* test, odds ratio for the Fisher's exact test, and Cramer's V for the likelihood ratio chi‐square test. For the correlations comparing reductions in depressive symptoms severity with changes in psychological functioning and cortical thickness, we used the beta values for within‐subject BDI covariates from the LME model to obtain the amount of change in psychological functioning and cortical thickness per reductions in BDI–II total scores by 10, which was comparable to overall reductions BDI–II total scores for the TBI‐plus‐depressive symptoms group. Similarly, we obtained the amount of change in functional connectivity per reduction in BDI–II total score by 10. Further, we calculated functional connectivity changes per Buckley cognitive factor scores of depressive symptoms by 4, which corresponded to the amount of reductions in Buckley cognitive factor scores for the BDI‐plus‐depressive symptoms group.

### 2.10 Control analyses

#### 2.10.1 Motion analysis

To identify whether there were systematic differences in participants’ head motion during rsfMRI scans, we performed LME analyses on (1) FD after motion censoring and trimming, and (2) on the percentage of motion censored volumes across scans.

#### 2.10.2 The assessment of the effects of injury characteristics

To assess the effects of initial injury severity on our findings, we repeated the LME analyses with an additional covariate: worst‐injury from the OSU TBI‐ID (2∼5). To identify potential effects of diverse post‐injury times on our findings, we also performed the LME analyses with the covariate of post‐injury time. Lastly, we divided the participants into mild and moderate/severe TBI groups and assessed whether there were group differences in reductions in BDI–II scores over time.

#### 2.10.3 Assessments of civilian versus veteran participants

We identified whether there were differences in initial TBI severity between civilians and veterans by performing a Fisher's exact test comparing the proportion of mild versus moderate/severe TBI among civilians and veterans. We also assessed the temporal changes in total BDI scores of veterans versus civilians. There was not an adequate sample size to allow for comparisons of the BDI covariates for civilians compared to veterans on psychological functioning scores, cortical thickness, or functional connectivity (e.g., *N* = 4 with rsfMRI scans for veterans within the TBI‐only group at TP_2_ [refer to Table S1]).

#### 2.10.4 The assessment of cortical thickness versus resting‐state functional connectivity

We obtained correlations between age‐adjusted, average cortical thickness of the four seed regions and corresponding seed‐based resting‐state functional connectivity at each time point. We then identified whether the spatial patterns of these correlations differed across time points and groups.

## 3 RESULTS

### 3.1 Demographics

All participants were in the chronic phase of TBI (∼9 years post‐injury on average). All demographics were matched at α = 0.05 (Table 1).

### 3.2 Reduced depressive symptoms severity following training

Average baseline BDI‐II scores for the TBI‐plus‐depressive symptoms group was approximately 24, corresponding to moderate depressive symptoms (Figure 1a). These baseline BDI–II scores were comparable to previous studies in psychological intervention for depression in TBI (Ashman et al., 2014; Bédard et al., 2014; D'Antonio et al., 2013). Baseline BDI–II scores for the TBI‐only group (7.0) was higher than those of healthy individuals (3.6) from a previous study (Han et al., 2015). The TBI‐plus‐depressive symptoms group showed a monotonic decrease in total BDI scores over time, relative to the TBI only group (*p *<* *.01). No statistically significant change in BDI scores was identified in the TBI‐only group (Figure 1a). Average BDI‐II scores for the TBI‐plus‐depressive symptoms group at the follow‐up stage was approximately 16, slightly greater than the threshold for depressive symptom (14). Reductions in total BDI scores were independent of training types (Figure 1b). Within the TBI‐plus‐depressive symptoms group, 16/43 participants (37%) right after training and 24/42 participants (57%) at the follow‐up reported minimal depressive symptoms (BDI_total_<14; Figure 1c,d). Subsequent Fisher's exact test on the proportion of SMART and BHW participants within the TBI‐plus‐depressive symptoms group revealed no statistically significant training‐specific effects at any time point at α = 0.05.

### 3.3 Improved psychological functioning versus reduced depressive symptoms severity

**Figure 2:**
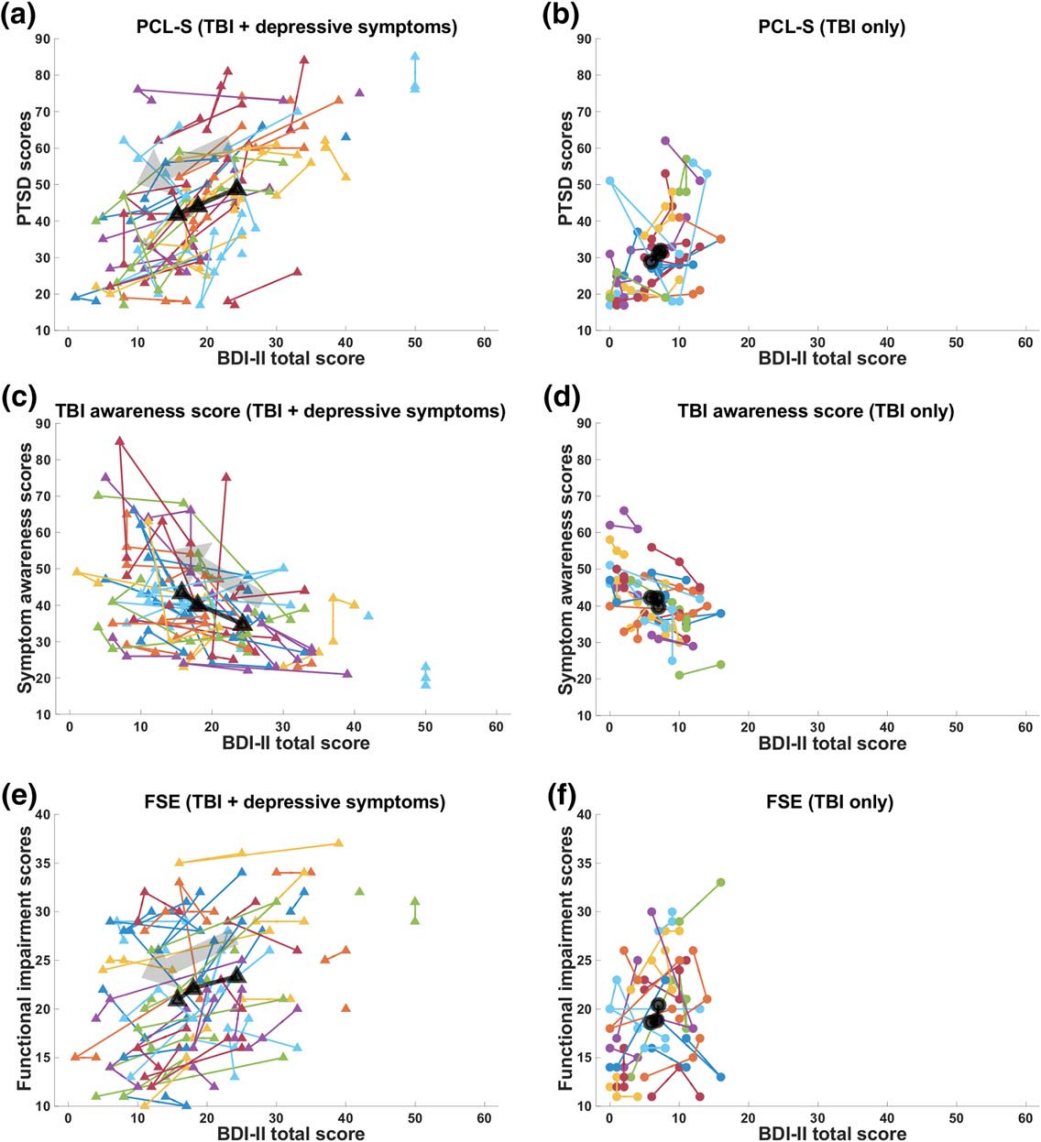
Trajectories of BDI‐II total scores versus psychological functioning scores. (a and b): BDI–II versus scores for PTSD symptoms from the PCL‐S. (c and d): BDI–II versus TBI awareness. (e and f): BDI‐II versus FSE. Each colored line represents the trajectory of each individual, and the black line represents group‐averaged trajectory. PCL‐S = posttraumatic stress disorder check‐list stressor‐specific; FSE = functional status examination [Color figure can be viewed at http://wileyonlinelibrary.com]

The average times between the assessment of psychological functioning were 9 (TP_2_) and 18 weeks (TP_3_) from baseline (Table 2). Within the TBI‐plus‐depressive symptoms group, statistically significant (*p *<* *.00625(=.05/8)) associations of reduced BDI_total_ scores over time co‐occurred with changes in scores from the PCL‐S, TBI awareness, and FSE (Table 3, Figure 2). Within the TBI‐only group, there were no statistically significant associations with any of the assessed psychological functioning scores at α = 0.00625 (Table 3, Figure 2).

**Table 3:** The effects of reduced BDI scores on improved psychological functioning scores

Psychological functioning scores	TBI‐plus‐depressive symptoms (*N* = 53)					TBI‐only (*N* = 26)				
	TP_1_	TP_2_	TP_3_	*p* values^a^	ES^b^	TP_1_	TP_2_	TP_3_	*p* values^a^	ES^b^
CW: Inhibition/switching (SS)	9.3 ± 3.3	10.0 ± 2.9	10.6 ± 2.9	>0.1	↑ 0.3	9.9 ± 3.3	10.3 ± 2.8	10.2 ± 3.4	>0.1	↓ 0.1
VF: Category switching, total correct (SS)	10.4 ± 3.3	10.4 ± 3.6	10.1 ± 3.7	>0.1	↑ 0.5	10.6 ± 3.9	10.2 ± 3.4	10.5 ± 3.4	>0.1	↓ 1.9
LM I: Immediate recall	12.8 ± 4.2	13.0 ± 3.5	14.7 ± 4.1	>0.1	↑ 1.0	13.2 ± 4.1	11.4 ± 3.5	13.8 ± 4.9	>0.1	↓ 1.8
LM II: Delayed recall	10.9 ± 4.7	11.6 ± 3.6	12.7 ± 5.3	>0.1	↑ 0.4	10.5 ± 5.2	9.8 ± 4.8	11.8 ± 4.8	>0.1	↓ 0.4
PCL‐S	48.9 ± 15.8	44.3 ± 18.4	42.0 ± 18.4	<0.001^*^	↓ 7.6	30.9 ± 11.4	32.0 ± 11.8	28.9 ± 12.4	>0.1	↓ 2.0
TBI awareness score	34.9 ± 8.8	40.3 ± 14.1	43.6 ± 13.8	<0.001^*^	↑ 5.4	39.7 ± 8.8	42.1 ± 8.2	42.3 ± 9.3	>0.1	↑ 3.9
FSE	23.4 ± 6.4	21.6 ± 7.7	21.0 ± 7.0	0.002^*^	↓ 1.6	20.4 ± 5.7	18.8 ± 5.7	18.6 ± 5.7	>0.1	↓ 1.8
Satisfaction with life scale	14.5 ± 6.8	16.9 ± 7.5	17.9 ± 6.9	0.01	↑ 2.2	23.4 ± 6.7	22.9 ± 6.8	24.4 ± 7.3	>0.1	↑ 1.8

a * represents *p *<* *.00625 (=.05/8).

b Changes in scores when reductions in total BDI scores by 10. “↑” and “↓” symbols indicates increases and decreases, respectively.

Abbreviations: CW = color‐word; VF = verbal fluency; LM = logical memory; BDI–II = Beck Depression Inventory‐II; PCL‐S = post‐traumatic stress disorder check list stressor‐specific; FSE = functional status examination; SS = scaled scores; ES = effect sizes; TP_1_ = within one month prior to training; TP_2_ = immediately after training completed; TP_3_ = 3 months after training completed.

### 3.4 Changes in cortical thickness versus reduced depressive symptoms severity

The QA procedures resulted in cortical thickness analysis for 143 structural MRI scans from 57 participants (TBI‐plus‐depressive symptoms group *n* = 36; Tables 2). The average times between MRI scans that passed QA procedures were 9 (TP_2_) and 21 weeks (TP_3_) from baseline (Table 2).

**Figure 3:**
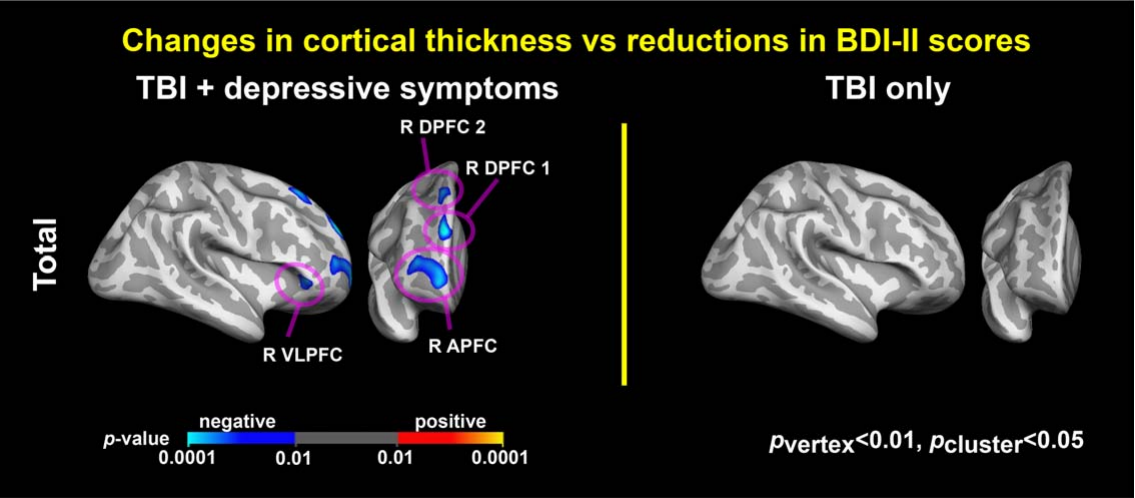
Associations between depressive symptoms and cortical thickness. Colormaps represent statistically significant associations of reduced BDI–II total scores with increased cortical thickness over time (*p*_vertex _< .01, *p*_cluster _< .05). R = right; VLPFC = ventrolateral prefrontal cortex; APFC = anterior prefrontal cortex; DPFC = dorsal prefrontal cortex [Color figure can be viewed at http://wileyonlinelibrary.com]

The whole‐brain LME analysis exhibited spatial patterns in regions where temporal changes in cortical thickness were associated with reduced BDI_total_ scores (Figure 3). Overall, only the TBI‐plus‐depressive symptoms group showed statistically significant (*p*_vertex _< .01, *p*_cluster _< .05) associations of change in depressive scores with temporal changes in cortical thickness. Specifically, regions with statistically significant associations included the right ventrolateral prefrontal cortex (VLPFC), anterior prefrontal cortex (APFC), and dorsal prefrontal cortex within Brodmann Area 9 (DPFC1) and Brodmann Area 8B (DPFC2). See Table 4 for coordinates and surface areas of these regions. The directionality of the associations over all four regions were negative, indicating that reduced total BDI scores following training were linearly correlated with increases in cortical thickness.

**Table 4:** Cortical thickness changes associated with reduced depressive symptoms severity following training

Index	ROI name	MNI coordinates (*x,y,z*) of center^a^	Surface area (mm^2^)^b^	Peak *p*_vertex_	*p*_cluster_
1	Right ventrolateral prefrontal cortex (BA 47)	(45.2, 31.4, −3.5)	252.1	<0.001	0.019
2	Right anterior prefrontal cortex (BA 10)	(24.0, 53.6, 4.8)	802.7	<0.001	<0.001
3	Right dorsal prefrontal cortex 1 (BA 9)	(12.6, 53.8, 30.2)	293.7	<0.001	0.008
4	Right dorsal prefrontal cortex 2 (BA 8B)	(16.3, 31.2, 53.9)	230.5	<0.001	0.031

a MNI coordinates correspond to a midpoint between pial and white matter surface.

b Surface area of white matter surface.

Abbreviation: MNI = Montreal Neurological Institute [Evans et al., 1993].

### 3.5 Changes in functional connectivity versus reduced depressive symptoms severity

**Figure 4:**
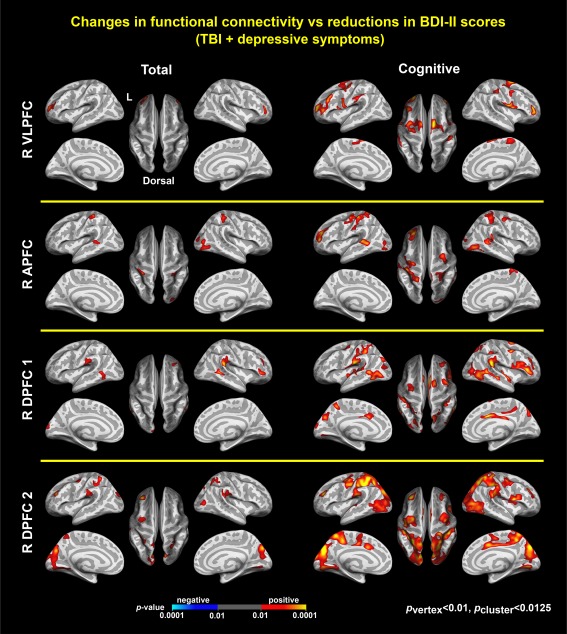
Associations between depressive symptoms and connectivity. Colormaps represent statistically significant associations of reduced BDI–II total scores (left) and Buckley cognitive factor scores of depressive symptoms (right) with reduced connectivity in R VLPFC, R APFC, R DPFC1, and R DPFC2 within the TBI‐plus‐depressive symptoms group, respectively (*p*_vertex _< .01, *p*_cluster _< .0125(=.05/4)). There were no statistically significant associations of reduced Buckley affective and somatic scores with increased connectivity. R = right; VLPFC = ventrolateral prefrontal cortex; APFC = anterior prefrontal cortex; DPFC = dorsal prefrontal cortex [Color figure can be viewed at http://wileyonlinelibrary.com]

The QA procedure (i.e., motion scrubbing) permitted rsFC analysis of 123 rsfMRI scans from 57 participants (TBI‐plus‐depressive symptoms group *n* = 36; Table 2). For the R VLPFC, R APFC, R DPFC1, and R DPFC2 seeds, statistically significant associations between changes in BDI_total_ scores and seed‐based connectivity occurred over multiple regions only within the TBI‐plus‐depressive symptoms group (Figure 4). Positive associations indicated that reductions in BDI_total_ scores following trainings were linearly correlated with decreases in functional connectivity.

Among the three Buckley factors, only changes in the cognitive factor scores in the TBI‐plus‐depressive symptoms group were associated with seed‐based connectivity for the R VLPFC, R APFC, R DPFC1, and R DPFC2 in multiple regions (Figure 4). These regions were inclusive of regions associated with BDI_total_ scores. Further, the patterns of associations with the cognitive factor were more prominent than those with the BDI_total_ scores. This suggests that reductions in symptoms related to the Buckley cognitive factor may be the primary driver of decreases in functional connectivity following cognitive training in chronic TBI.

### 3.6 The effect sizes for neural correlates with reduced depressive symptoms severity

**Figure 5:**
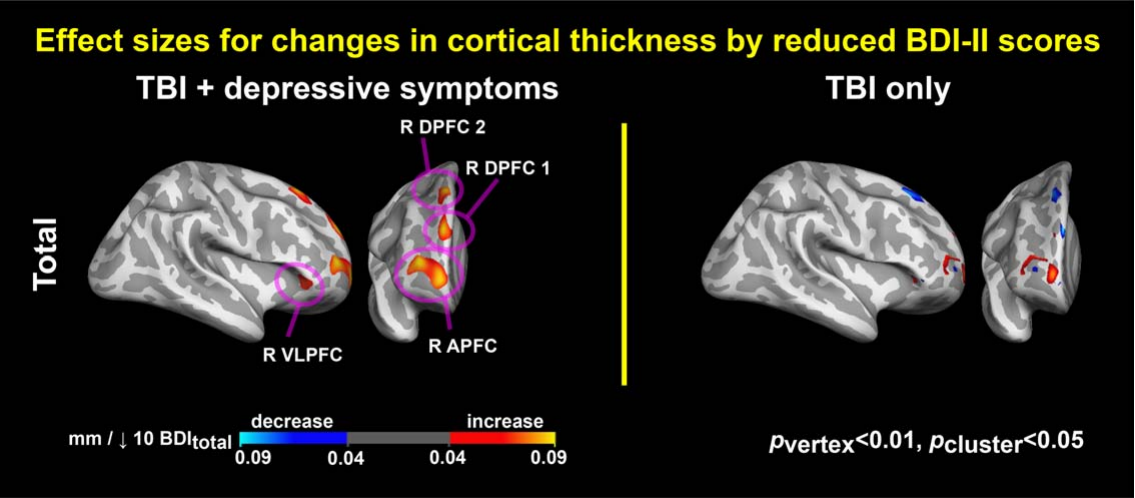
Effect sizes for associations between changes in cortical thickness and reductions in depressive symptoms. Colormaps represent increases or decreases in cortical thickness per reductions in BDI–II total scores by 10. R = right; VLPFC = ventrolateral prefrontal cortex; APFC = anterior prefrontal cortex; DPFC = dorsal prefrontal cortex [Color figure can be viewed at http://wileyonlinelibrary.com]

**Figure 6:**
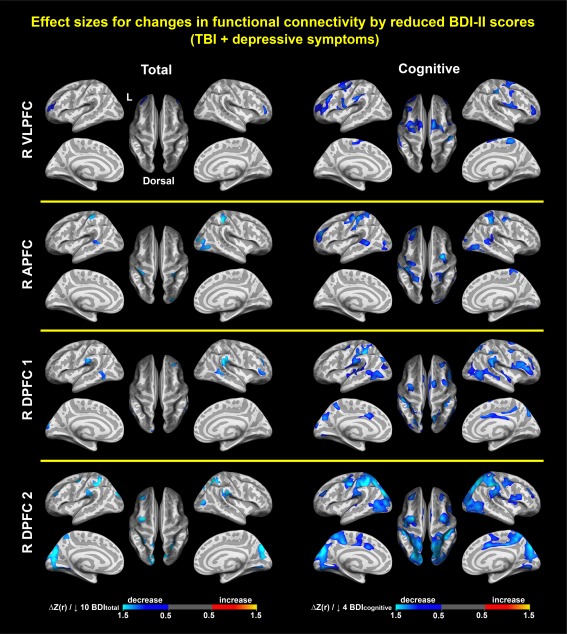
Effect sizes for associations between changes in functional connectivity and reductions in depressive symptoms. Colormaps represent increases or decreases in functional connectivity per reductions in BDI–II total scores by 10 (left) and Buckley cognitive factor scores of depressive symptoms by 4 (right). R = right; VLPFC = ventrolateral prefrontal cortex; APFC = anterior prefrontal cortex; DPFC = dorsal prefrontal cortex [Color figure can be viewed at http://wileyonlinelibrary.com]

When BDI‐II total scores for the TBI‐plus‐depressive symptoms were reduced by 10, cortical thickness was increased by 0.04–0.09 mm (Figure 5), and z‐scores for functional connectivity were decreased by 0.5–1.5 (Figure 6 *left*). When Buckley cognitive factor scores for the TBI‐plus‐depressive symptoms were reduced by 4, *Z*‐scores for functional connectivity were decreased by 0.5–1.5 (Figure 6 *right*).

### 3.7 Control analyses results

#### 3.7.1 Motion analysis results

There were no statistically significant group differences in trajectories of the percentage of motion censored volumes or FD after censoring and trimming at α = 0.05 (Supporting Information Table S2).

#### 3.7.2 The effects of injury characteristics

The inclusion of covariates for initial injury severity or post‐injury time did not alter the observed patterns of (1) BDI changes over time (Supporting Information Figure S1) or (2) associations between changes in BDI scores and improvement in psychological functioning scores (Table S3). The uncorrected (*p*_vertex _< .01) maps for correlates of changes in cortical thickness and BDI scores were not affected by inclusion of covariates for initial injury severity or post‐injury time (Supporting Information Figure S2). Compared to uncorrected (*p*_vertex _< .01) maps for the LME analysis on rsFC (Supporting Information Figure S3), the inclusion of covariates for initial injury severity (Supporting Information Figure S4) or post‐injury (Supporting Information Figure S5) did not alter the main findings. When we divided participants into mild and moderate/severe TBI groups, the mild TBI group showed higher baseline BDI–II scores (*p *=* *.02), which resulted in marginal (*p *=* *.06) group differences in reductions in BDI–II scores over time (Supporting Information Figure S6a). This is consistent with other investigations that have found higher levels of depression in mild TBI compared to moderate and severe TBI (Homaifar et al., 2009; Rowland, Lam, & Leahy, 2005).

#### 3.7.3 Civilians versus veterans

The civilian participant group was comprised of 33 mild TBI and 20 moderate/severe TBI cases. The veteran participant group was comprised of 20 mild TBI and 6 moderate/severe TBI cases. There were no statistically significant differences in the proportion of mild and moderate/severe TBI between civilians and veterans at *α *=* *.05. The veteran participants had higher baseline depressive symptoms severity than the civilian participants (*p *<* *.01). However, both civilians and veterans showed reductions in scores for depressive symptoms severity, yielding no statistically significant group‐by‐time interactions at *α *=* *.05 (Supporting Information Figure S6b).

#### 3.7.4 Correlations between cortical thickness and resting‐state functional connectivity

There were correlations between cortical thickness and resting‐state connectivity (*p*_vertex _< .01, *p*_cluster _< .05). The spatial patterns of these correlations differed across time points and groups (Supporting Information Figures S7–S9)

## 4 DISCUSSION

We demonstrated reduced depressive symptom severity following cognitive training to address chronic TBI symptoms, its impact on improvement in psychological functioning, and its correlation to changes in cortical thickness and resting‐state functional connectivity. To our knowledge, this is the first study to report neural plasticity associated with reduced depressive symptom severity following cognitive intervention for TBI. Strengths of our study include our study design (instructors were blinded to the presence of depressive symptoms in participants, and participants were unaware of the type of intervention they were in) and the methodology that allowed us to identify the effects of time‐varying covariates (i.e., BDI scores) on other time‐varying measures (e.g., rsFC).

### 4.1 Reduction in depressive symptom severity

The TBI‐plus‐depressive symptoms group showed reduced BDI‐II scores over time relative to the controls (Figure 1a), consistent with previous studies in the remediation of depression following training in individuals after TBI (Ashman et al., 2014; D'Antonio et al., 2013; Fann et al., 2015; Ponsford et al., 2016; Tiersky et al., 2005; Topolovec‐Vranic et al., 2010). A weakness of several prior studies has been the lack of control groups (Fann et al., 2009; Gertler et al., 2015). We utilized an active control group (i.e., the TBI‐only group) who underwent the same intervention programs and assessed group‐by‐time interaction effects—the gold standard for training‐induced changes. This feature of the design helps to address the test‐retest reliability of BDI‐II scores. Note that the TBI‐only group unlikely experienced ‘floor effects’ because of (1) higher baseline BDI–II scores than healthy non‐TBI individuals in a previous study (7.0 vs. 3.6) (Han et al., 2015) and (2) slight monotonic reductions in BDI‐II scores over time (7.0 at TP_1_ vs. 5.9 at TP_3_).

Reductions in depressive symptoms severity following cognitive intervention were independent of the employed intervention program (Figure 1b,d). This phenomenon is consistent with other studies that administered multiple psychological intervention programs for comorbid depression in TBI (Ashman et al., 2014; D'Antonio et al., 2013; Ponsford et al., 2016). The strong inclusion of social interactions in our training programs may explain the reductions in depressive symptoms in TBI, as previous studies reported that the effects of supportive psychotherapy promoting participants’ social interactions with instructors and cognitive behavioral therapy were comparable (Ashman et al., 2014; D'Antonio et al., 2013).

### 4.2 Improvement in psychological functioning

Within the TBI‐plus‐depressive symptoms group, reductions in depressive symptom severity following cognitive intervention were associated with improvements in PTSD‐related symptoms severity, the level of self‐perceived disability relative to the pre‐injury state (i.e., TBI awareness score), and functional status (Table 3). This may not be surprising, as several studies in TBI reported that BDI scores are associated with PTSD scores from the PCL (Esterman et al., 2013; Han et al., 2016), TBI awareness (Malec, Brown, Moessner, Stump, & Monahan, 2010), and self‐reported functioning from the FSE (Hudak et al., 2012) at a single time point. Our findings extend this line of research by demonstrating the tight coupling of these measures not only at a single time point, but also in the context of changes that can be observed over multiple time points accompanying improvement.

### 4.3 Changes in cortical thickness

Reduced depressive symptom severity after cognitive training for TBI was related to increased cortical thickness over time only within the TBI‐plus‐depressive symptoms group (Figure 3). The depression literature has reported increases in cortical thickness following treatment for depression (van Eijndhoven et al., 2016; Phillips, Batten, Tremblay, Aldosary, & Blier, 2015; Pirnia et al., 2016). Our study extends this line of research by demonstrating intervention‐induced neuroplasticity in TBI with comorbid depressive symptoms. Previous studies demonstrated reduced cortical thinning in individuals with major depression (Peterson et al., 2009; Schmaal et al., 2017) and depression plus blunt TBI (Mollica et al., 2009). These previous studies support the observed increases in cortical thickness following intervention in current study.

Increases in cortical thickness occurred in the prefrontal cortex, which is associated with controlling emotional responses reflected in limbic activity (Ochsner & Gross, 2005). The imbalance of brain activity between the prefrontal cortex and limbic regions relates to the cognitive (i.e., emotion regulation) aspects of depression (DeRubeis et al., 2008). As such, several studies reported reduced cortical thickness of the prefrontal cortex in individuals with depression (van Tol et al., 2014; Tu et al., 2012) and in individuals with depression plus blunt TBI (Mollica et al., 2009). Thus, thickened prefrontal cortex after an intervention, as seen in this study, may be related to improved emotion regulation in TBI.

### 4.4 Changes in functional connectivity

Within the TBI‐plus‐depressive symptoms group, training‐induced reductions in depressive symptoms severity was associated with reduced rsFC over time (Figure 4). As with previous reports describing changes in rsFC following pharmacological treatments (Li et al., 2013), electroconvulsive therapy (Leaver et al., 2016), and cognitive behavioral therapy (Chattopadhyay et al., 2017), our findings demonstrated the potential utility of rsFC for identifying neuroimaging markers associated with reduced depressive symptoms following interventions. Previously, we reported elevated amgydala connectivity in TBI individuals with depressive symptoms, relative to healthy individuals (Han et al., 2015). Thus, the positive association between rsFC and depressive symptom severity indicates that cognitive training restored elevated rsFC by TBI and depression to healthier status.

Among the three Buckley BDI factors, only changes in the cognitive factor for the TBI‐plus‐depressive symptoms group were associated with training‐induced neural plasticity of rsFC (Figure 4). This finding exhibited the specificity of rsFC with regard to subtypes of depressive symptoms in TBI. Further, there were more prominent patterns of neural correlates with the cognitive aspect of depressive symptoms (i.e., the Buckley cognitive factor) than with total scores (Figure 4). This suggests that reductions in the Buckley cognitive factor score led to neural correlates of reduced overall depressive symptoms severity after cognitive training. The Buckley cognitive factor includes items related to sadness, pessimism, past failure, guilt, punishment feelings, self‐dislike, self‐criticalness, suicidal ideation, and worthlessness. Most of these items reflect negative self‐perceptions. Thus, more positive self‐perceptions following group‐based cognitive training primarily contributed to the observed training‐induced neuroplasticity in rsFC.

### 4.5 Explaining reduced depressive symptom severity

First, social interactions during small‐group‐based interventions might have reduced depressive symptom severity. Previous studies reported positive effects of increased social activity and group‐based cognitive behavioral therapy on depressive symptoms (Ashman et al., 2014; Cruwys et al., 2013; Cruwys et al., 2014). Further, older participants enrolled in a social service program aimed at improving memory and executive function through social engagement demonstrated increases in brain activity in the prefrontal cortex relative to controls (Carlson et al., 2009). The observed neural correlates of reduced depressive symptoms in prefrontal regions (Figures 3 and 4) may be relevant in this regard. The frontopolar region is involved in social judgment and behavior (Cicerone, Levin, Malec, Stuss, & Whyte, 2006), and thinner cortex in this region leads to problematic social behavior of children with TBI (Levan et al., 2016). The VLPFC is associated with social exclusion (Eisenberger, Lieberman, & Williams, 2003) and has been linked to social problem‐solving capability (Barbey et al., 2014).

Second, improved emotion regulation after cognitive training might lead to reduced depressive symptom severity. Cognitive behavioral therapy is thought to stabilize brain activity between the prefrontal cortex and limbic regions by improving inhibitory function of the prefrontal cortex (DeRubeis et al., 2008). Thickened prefrontal cortex (Figure 3) along with reduced connectivity between dorsal prefrontal cortex and cingulate cortex (Figure 4), as seen in the current study, may reflect improved emotion regulation following cognitive training.

Third, improved cognitive functioning following training may have contributed to reductions in related depressive symptoms. TBI‐induced cognitive impairment is associated with depressive symptoms (Rapoport et al., 2005), and cognitive training for depression improves cognitive functions (Motter et al., 2016; Tiersky et al., 2005). Our TBI participants with depressive symptoms showed improvement in cognitive and daily‐life functioning, regardless of training group, and this improvement was associated with reduced depressive symptoms (Table 3).

Note that all these factors remain speculative without direct evidence. Thus, other factors might contribute to reduced depressive symptom severity in this report.

### 4.6 Limitations and future directions

None of the participants sought medical attention for depression prior to the current study, although the TBI‐plus‐depressive symptoms group reported mild‐to‐severe depressive symptoms according to the BDI manual (Beck et al., 1996). Whereas previous studies demonstrated that the BDI–II is a reliable and valid measure and group‐by‐time analyses in current study support the test‐retest reliability of it, the BDI‐II is a self‐report measure of depression. This could lead to bias in the severity level reported in this population (Malec et al., 2007).

Similar to several studies investigating the treatment for depression after TBI (Fann et al., 2009), addressing depression was not the primary purpose of our cognitive intervention programs. We prospectively included depressive symptoms severity as one of the outcome measures in our clinical trial, but the primary purpose of the two interventions was to improve cognitive functioning by learning strategy‐based reasoning skills or new information about the brain. Thus, we interpreted reduced depressive symptom severity after intervention as secondary effects.

Unlike our previous report (Vas et al., 2016), we did not find training‐specific effects on depressive symptoms. Apparent discrepancy may be attributable to different sample inclusion criteria, heterogeneity, and sample sizes. Additionally, we did not assess neural correlates of reduced depressive symptoms in subcortical regions. Based on reported alterations in subcortical regions in depression (Mayberg, 1997), future directions include the assessment of subcortical regions in our participants.

## 5 CONCLUSION

We demonstrated reduced overall depressive symptom severity following cognitive interventions for chronic TBI and its correlation to psychological functioning score dynamics, cortical thickness, and rsFC. Intervention‐induced reductions in rsFC were associated with the Buckley cognitive factor (related to self‐perception) of the BDI only among the three Buckley factors. Our findings suggest that cortical thickness and rsFC may be promising biomarkers sensitive to evaluating reductions in depressive symptom severity following cognitive intervention for chronic TBI.

## Supporting information

Additional Supporting Information may be found online in the supporting information tab for this article.

Supporting Information
