# Supplementary material for: Neural correlates of reduced depressive symptoms following cognitive training for chronic traumatic brain injury
Source: Hum Brain Mapp. 2018 Mar 23;39(7):2955–71. doi: 10.1002/hbm.24052 (PMC6055759; doi:10.1002/hbm.24052)
Supplement: Supplementary file 1 — Supporting Information [file HBM-39-2955-s001.pdf]

Supplementary Material

Title: Neural correlates of reduced depressive symptoms following cognitive training for chronic traumatic brain injury[†]

Authors: Kihwan Han[1,*], David Martinez[1], Sandra B Chapman[1], Daniel C Krawczyk[1,2]

Affiliations:

[a]Center for BrainHealth®, School of Behavioral and Brain Sciences, The University of Texas at Dallas

[b]Department of Psychiatry, University of Texas Southwestern Medical Center

Corresponding author: Kihwan Han, PhD

Center for BrainHealth®

School of Behavioral and Brain Sciences

2200 West Mockingbird Lane, Mail Stop: CBH

Dallas, TX 75235

USA

Email: kihwan.han98@gmail.com

Tel: 1-972-883-3429

Fax: 1-214-905-3026

Running title: Neuroplasticity of alleviated depression in TBI

[†]The views and opinions expressed in this article are those of the authors and do not reflect the official policy or position of the Department of the Army, Department of the Air Force, Department of Defense or United States Government.

Supplementary Table

Table S1. The sample size (civilians, veterans) of psychological functioning assessments and MRI scans per time point by group.

Data type	Time point	TBI-plus-depressive symptoms	TBI-only
Psychological functioning scores	TP_1	53 (34, 19)	26 (19, 7)
	TP_2	43 (27, 16)	24 (17, 7)
	TP_3	42 (26, 16)	22 (16, 6)
Structural MRI scans[a]	TP_1	36 (23, 13)	21 (15, 6)
	TP_2	30 (20, 10)	17 (12, 5)
	TP_3	23 (15, 8)	16 (10, 6)
Resting-state fMRI scans[a]	TP_1	30 (19, 11)	18 (13, 5)
	TP_2	26 (17, 9)	15 (11, 4)
	TP_3	20 (14, 6)	14 (9, 5)

Note: See Table 2 for the abbreviations.

[a] Only MRI scans that passed the quality assurance procedures were reported.

Table S2. The amount of subject motion during fMRI scans per time point by group

Measures	TBI-plus-depressive symptoms (N=36)			TBI-only (N=21)			p <0.05
	TP_1	TP_2	TP_3	TP_1	TP_2	TP_3	
Motion censored volumes (%)	18.8 ± 15.2	17.5 ± 13.3	18.6 ± 14.8	13.4 ± 9.0	12.2 ± 11.1	17.7 ± 16.8	none
FD after censoring and trimming (mm)	0.17 ± 0.04	0.17 ± 0.05	0.15 ± 0.04	0.15 ± 0.04	0.15 ± 0.06	0.16 ± 0.06	none

Table S3. The effects of reduced BDI scores on improved neuropsychological assessment scores, adjusted for initial injury severity and post-injury time

Neuropsychological measures	TBI-plus-depressive symptoms (N=53)		TBI-only (N=26)	
	With injury severity covariate[a]	With post-injury time covariate[a]	With injury severity covariate[a]	With post-injury time covariate[a]
CW: Inhibition/switching (SS)	>0.1	>0.1	>0.1	>0.1
VF: Category switching, total correct (SS)	>0.1	>0.1	>0.1	>0.1
LM I: Immediate recall	>0.1	>0.1	>0.1	>0.1
LM II: Delayed recall	>0.1	>0.1	>0.1	>0.1
PCL-S	<0.001*	<0.001*	>0.1	>0.1
TBI awareness score	<0.001*	<0.001*	>0.1	>0.1
FSE	0.002*	0.002*	>0.1	>0.1
Satisfaction with life scale	0.01	0.01	>0.1	>0.1

Note: See Tables 2, 3 for the abbreviations.

[a] p-values; * represents $p < 0.00625$ (=0.05/8).

Supplementary Figures

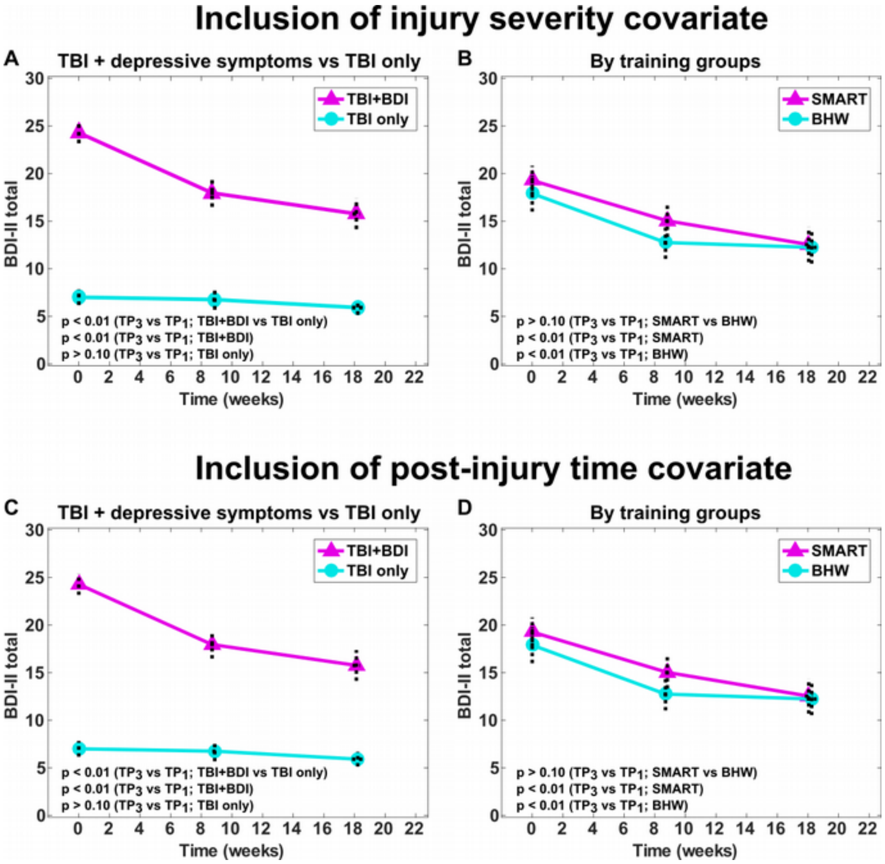

Fig. S1. The effects of injury profiles on depressive symptoms. The plots represent temporal changes in depressive symptoms severity according to the presence of depressive symptoms and types of intervention programs, adjusted for initial injury severity (A-B) and post-injury time (C-D), respectively.

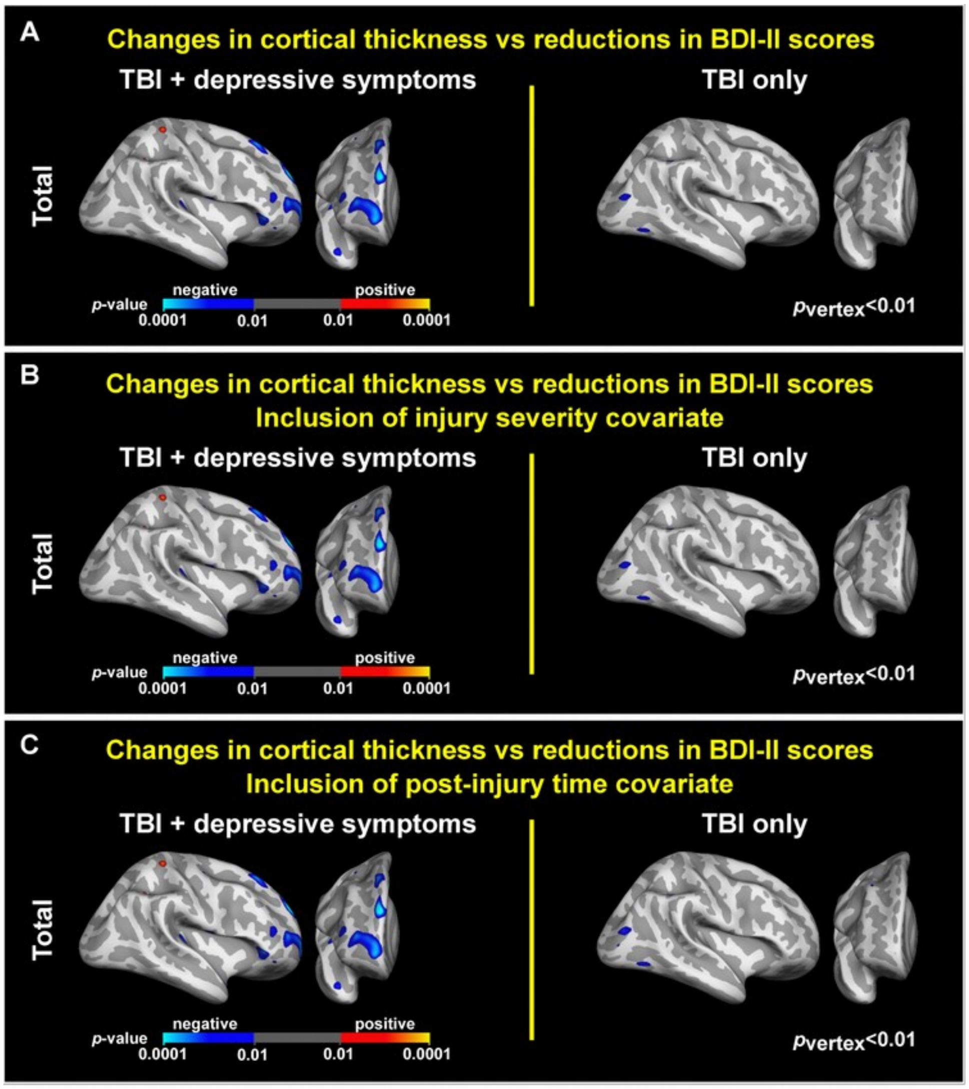

Fig. S2. The effects of injury profiles on depressive symptoms versus cortical thickness. Colormaps represent the associations of reduced BDI-II total scores with increased cortical thickness over time at p_{vertex}<0.01 (A) and those adjusted for initial injury severity (B) and post-injury time (C). See Fig. 3 for the abbreviations, respectively.

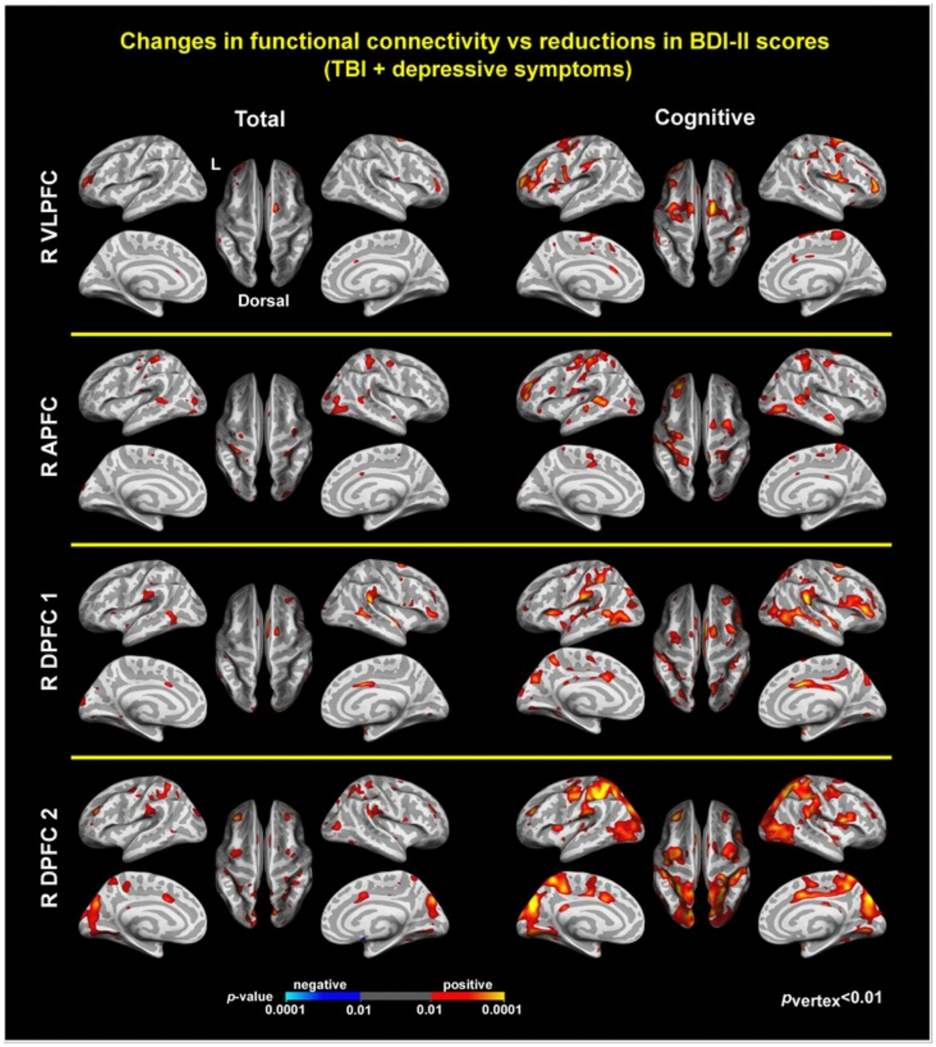

Fig. S3. Uncorrected results of depressive symptoms versus connectivity. Colormaps represents the associations of reduced BDI-II total scores (left) and Buckley cognitive factor scores (right) with reduced connectivity in R VLPFC, R APFC, R DPFC1, and R DPFC2 within the TBI-plus-depressive symptoms group, respectively (p_{vertex}<0.01). See Figs. 3 for the abbreviations.

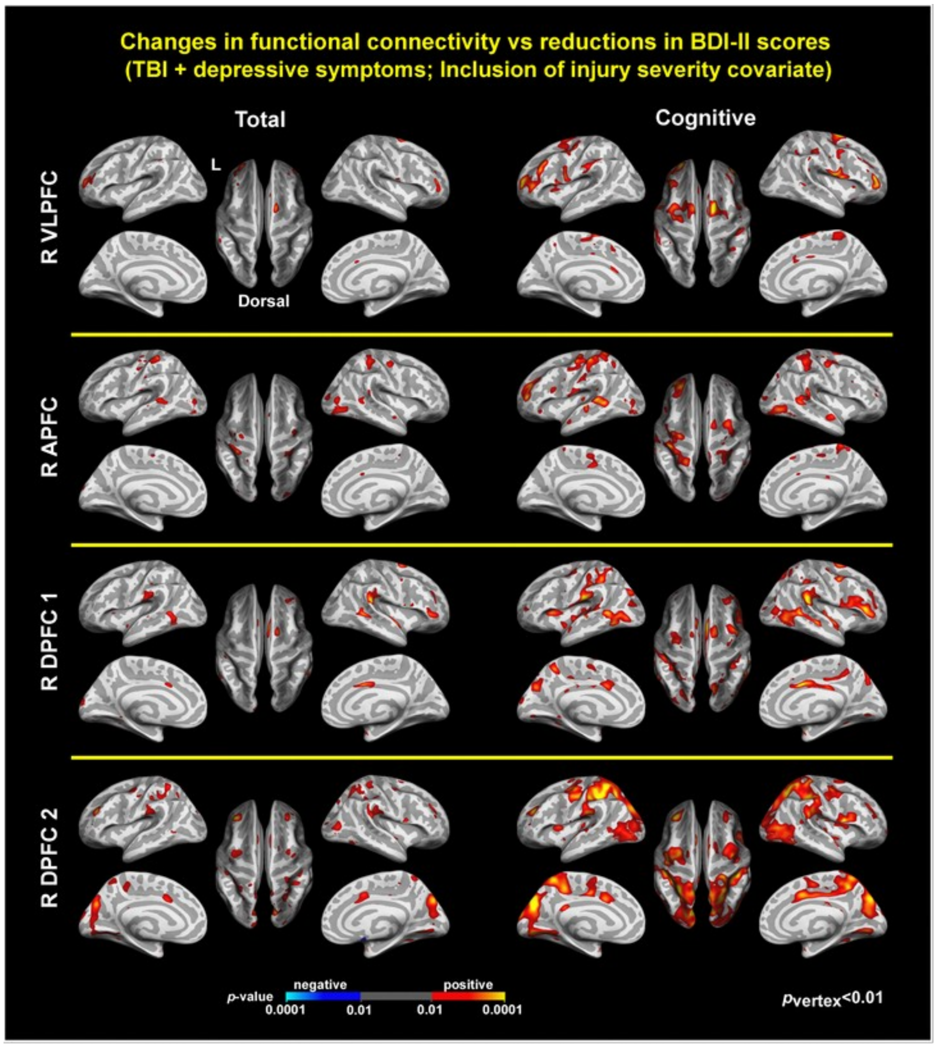

Fig. S4. The effects of injury severity on depressive symptoms versus connectivity. Colormaps represent the associations of reduced BDI-II total scores (left) and Buckley cognitive factor scores (right) with reduced connectivity in R VLPFC, R APFC, R DPFC1, and R DPFC2 within the TBI-plus-depressive symptoms group, respectively ($p_{\text{vertex}}<0.01$). The maps were adjusted for initial injury severity. See Figs. 3 for the abbreviations.

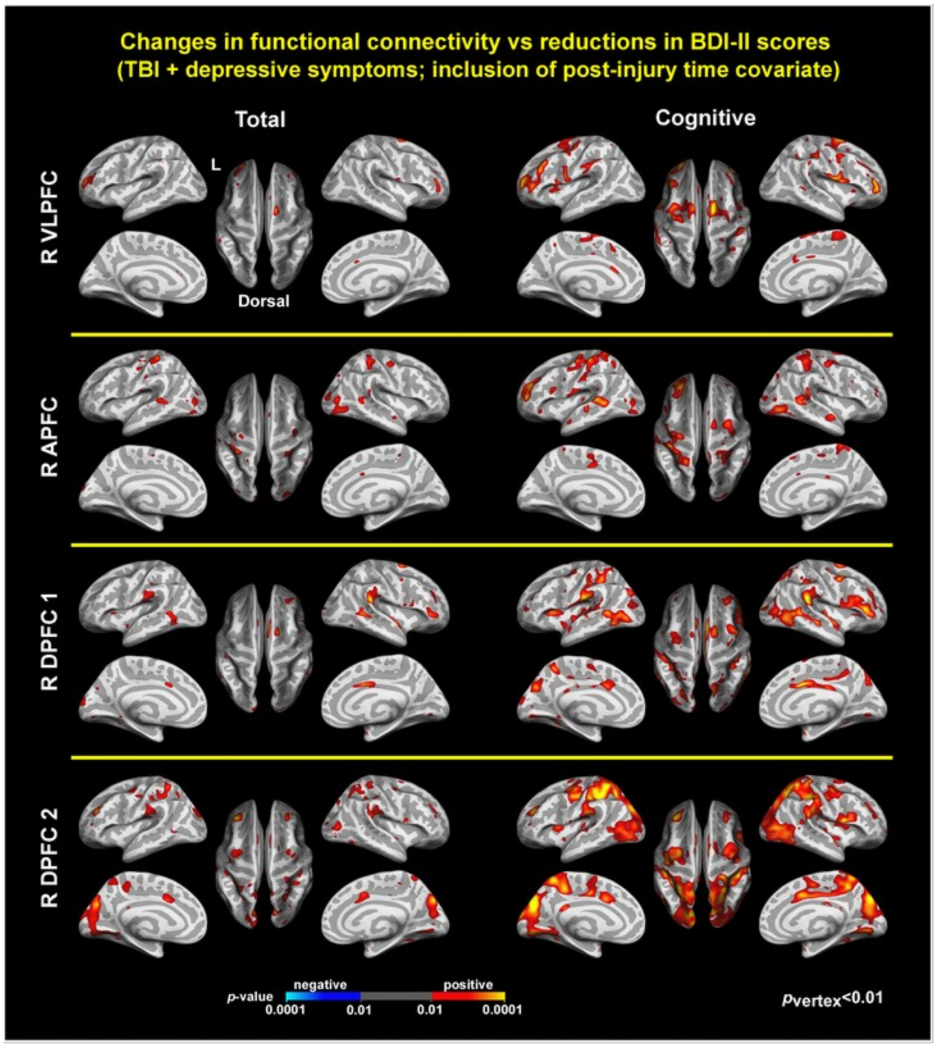

Fig. S5. The effects of post-injury time on depressive symptoms versus connectivity. Colormaps represent the associations of reduced BDI-II total scores (left) and Buckley cognitive factor scores (right) with reduced connectivity in R VLPFC, R APFC, R DPFC1, and R DPFC2 within the TBI-plus-depressive symptoms group, respectively (p_{vertex}<0.01). The maps were adjusted for post-injury time. See Figs. 3 for the abbreviations.

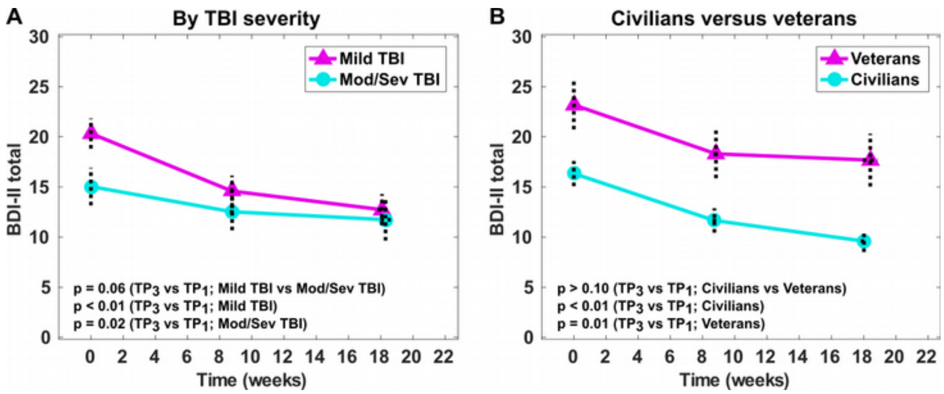

Fig. S6. Temporal changes in depressive symptoms. (A) Mild versus moderate/severe TBI (B) Civilians versus veterans.

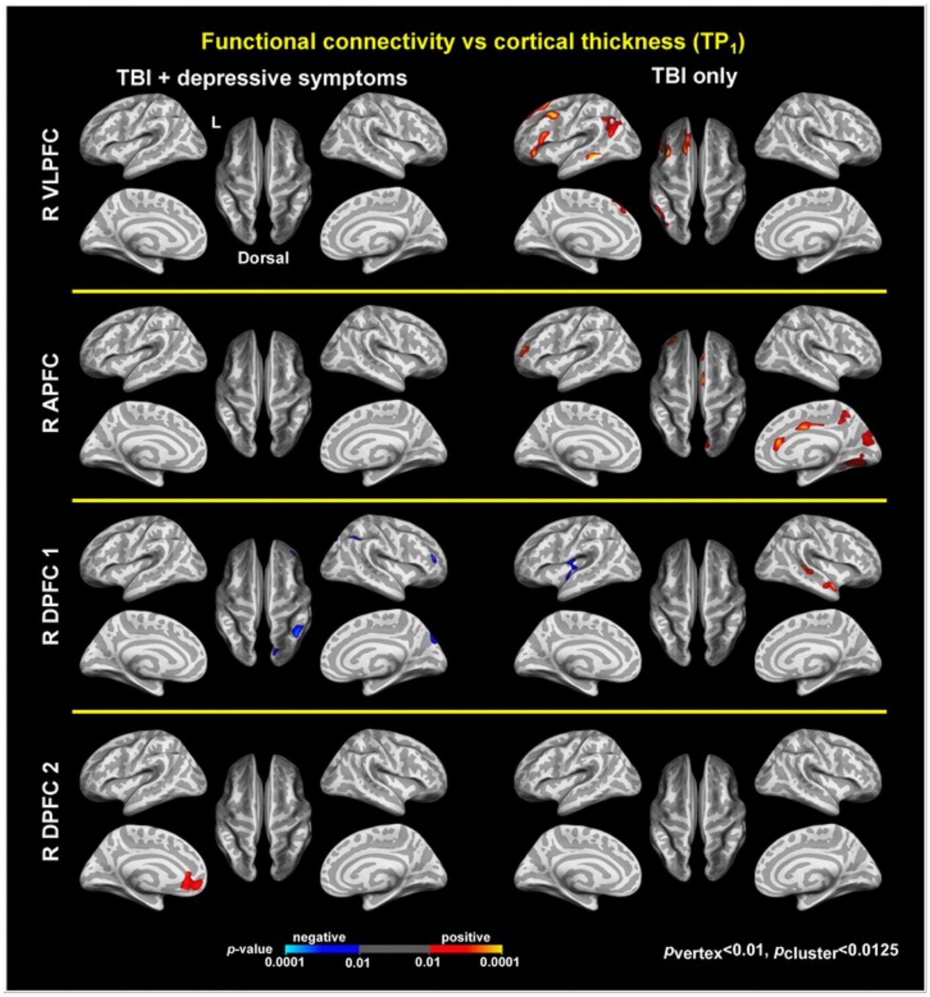

Fig. S7. Correlations between age-adjusted, average cortical thickness of seed regions and corresponding seed-based functional connectivity at TP_1.

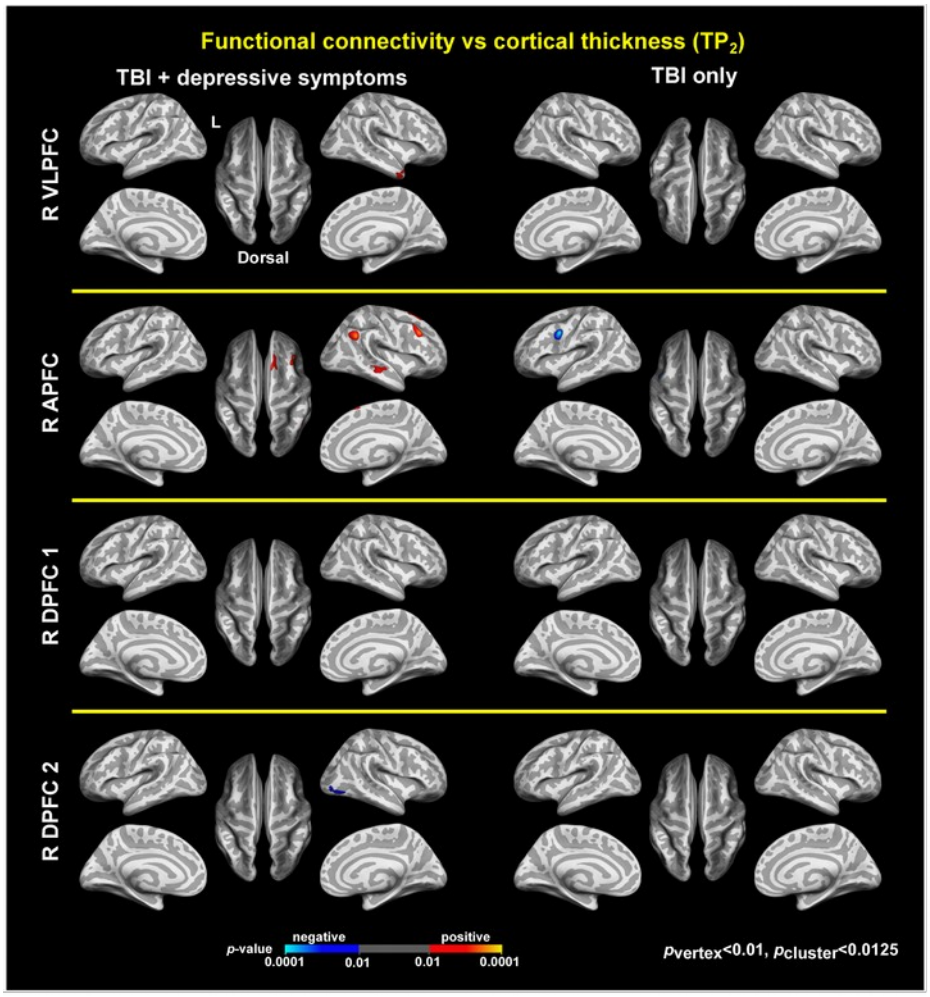

Fig. S8. Correlations between age-adjusted, average cortical thickness of seed regions and corresponding seed-based functional connectivity at TP_2.

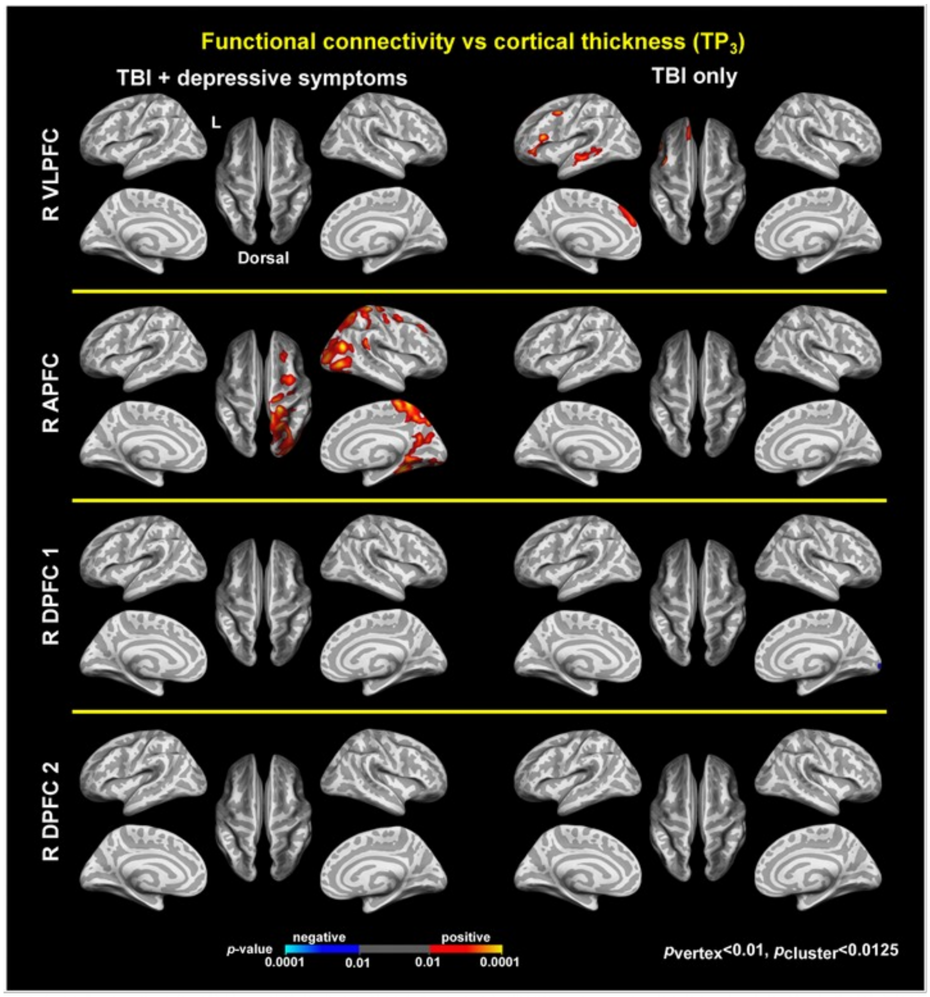

Fig. S9. Correlations between age-adjusted, average cortical thickness of seed regions and corresponding seed-based functional connectivity at TP_3.
